# Sustained Clinical Efficacy and Mucosal Healing of Thiopurine Maintenance Treatment in Ulcerative Colitis: A Real-Life Study

**DOI:** 10.1155/2018/4195968

**Published:** 2018-10-03

**Authors:** Daniela Pugliese, Annalisa Aratari, Stefano Festa, Pietro Manuel Ferraro, Rita Monterubbianesi, Luisa Guidi, Maria Lia Scribano, Claudio Papi, Alessandro Armuzzi

**Affiliations:** ^1^IBD Unit, Presidio Columbus Fondazione Policlinico Universitario A. Gemelli IRCCS Università Cattolica, Rome 00168, Italy; ^2^IBD Unit, S. Filippo Neri Hospital, Rome 00135, Italy; ^3^Nephrology, Presidio Columbus Fondazione Policlinico Universitario A. Gemelli IRCCS Università Cattolica, Rome 00168, Italy; ^4^IBD Unit, San Camillo Forlanini Hospital, Rome 00152, Italy

## Abstract

**Background and Aims:**

Thiopurines are commonly used for treating ulcerative colitis (UC), despite the fact that controlled evidence supporting their efficacy is limited. The aim of this study was to evaluate the long-term outcome of thiopurines as maintenance therapy in a large cohort of UC patients.

**Methods:**

All UC patients receiving thiopurine monotherapy at three tertiary IBD centers from 1995 to 2015 were identified. The primary endpoint was steroid-free clinical remission. Secondary endpoints were mucosal healing (MH), defined as Mayo endoscopic subscore 0, long-term safety, and predictors of sustained clinical remission.

**Results:**

We identified 192 patients, contributing a total of 747 person-years of follow-up (median follow-up 36 months, range 1–210 months). Steroid dependency was the most common indication for thiopurine treatment (58%). Steroid-free remission occurred in 45.3% of patients; 36.3% stopped thiopurines because of treatment failure and 18.2% for adverse events or intolerance. The cumulative probability of maintaining steroid-free remission while on thiopurine treatment was 87%, 76%, 67.6%, and 53.4% at 12, 24, 36, and 60 months, respectively. MH occurred in 57.9% of patients after a median of 18 months (range 5–96). No independent predictors of sustained clinical remission could be identified.

**Conclusions:**

Thiopurines represent an effective and safe long-term maintenance therapy for UC patients.

## 1. Introduction

Ulcerative colitis (UC) is an inflammatory bowel disease (IBD) needing chronic maintenance therapies in order to prevent symptom relapses and disease progression [1]. Aminosalicylates are the first-line medical option for remission maintenance in the long term for mild to moderate disease [2, 3]. Nevertheless, after a moderate-to-severe disease flare requiring systemic corticosteroids, up to 20% of patients need to escalate therapies because of the development of steroid-dependency and approximately 15% because of steroid-refractoriness [4]. Thiopurines, azathioprine (AZA), and 6-mercaptopurine (6MP) have been considered the reference maintenance treatment for patients with steroid-dependent and steroid-refractory moderate-to-severe UC for many years and are recommended as the first line immunosuppressive therapy by major guidelines [1, 5].

Controlled data supporting the efficacy of thiopurines in UC are limited and are not as robust as in Crohn's disease (CD) [6, 7]. Few old randomized controlled trials (RCTs) addressing AZA and 6MP for the treatment of UC have relevant methodological limitations such as small sample size, inadequate thiopurine dose, heterogeneity of patient populations, limited follow-up, and not well-defined endpoints [8–14]. Despite these limitations, a systematic review and meta-analysis addressing the use of thiopurines in UC concluded that AZA and 6MP are more effective than placebo for the prevention of relapse in UC, with a number needed to treat (NNT) of 5 and an absolute risk reduction (ARR) of 23% compared to placebo [15]. Moreover, the efficacy of thiopurines in UC is supported by several uncontrolled observational studies: a mean efficacy of 65% and 75% for remission induction and maintenance, respectively, has been reported [15]. However, study designs, patients' characteristics, length of follow-up, and endpoints considered are very heterogeneous across studies, making robust conclusions very challenging. Furthermore, mucosal healing (MH) in UC has been poorly investigated with thiopurines, despite the fact that MH has recently emerged as a therapeutic goal in the management of IBDs, both for clinical trials and clinical practice [16].

The aim of this study is to evaluate the long-term effectiveness of thiopurines for maintaining clinical and endoscopic remissions in a large cohort of UC patients in a real-life setting and to explore possible predictors of sustained effectiveness.

## 2. Patients and Methods

This is an open-label retrospective study of consecutive UC patients treated with thiopurines at three IBD referral centers in Rome, Italy (Presidio Columbus, Fondazione Policlinico Universitario A. Gemelli IRCCS Università Cattolica del Sacro Cuore; S. Filippo Neri Hospital; and San Camillo Forlanini Hospital). Eligible patients included men and women older than 18 years with an established diagnosis of UC, who received maintenance treatment with thiopurine monotherapy from 1995 to 2015. Patients receiving thiopurine monotherapy after a course of anti–tumour necrosis factor (TNF) alpha treatment or after rescue therapy with cyclosporine for severe steroid refractory UC were excluded.

A shared common database was used to collect demographic and clinical data. The following variables were recorded: age at diagnosis, gender, disease duration, disease extent, endoscopic activity, smoking habit, indication for thiopurine treatment, type of thiopurines used (AZA or 6MP), and concomitant medications during induction and maintenance phases. The indications for thiopurine therapy were classified as the following: (1) steroid dependence, (2) maintenance therapy after a severe acute attack responsive to intravenous (iv) steroids, and (3) maintenance therapy for patients with mild to moderate disease with frequent relapses despite optimized treatment with aminosalicylates. Steroid dependency was defined according to the Italian Group for the Study of IBD (IG-IBD) guidelines [5] or as need of at least two steroid courses in the previous year. Patients with two or more clinical relapses in the last year despite appropriate oral and rectal aminosalicylates were considered having frequent relapses. Disease extent was defined according to the Montreal classification [17]; endoscopic activity was evaluated according to the Mayo endoscopic subscore [18]. Baseline endoscopy had to be performed within 3 months before starting thiopurines; follow-up endoscopies were scheduled at variable time points according to clinical judgment. MH was defined as Mayo endoscopic subscore of 0 and assessed for patient achieving sustained steroid-free clinical remission [18]. At the last follow-up visit, data regarding disease activity and whether patients were still on thiopurine maintenance were recorded. The reasons for discontinuation of thiopurines were classified as (1) sustained steroid-free clinical remission; (2) thiopurine failure, defined as clinical relapse requiring therapeutic escalation with corticosteroids and/or biologics or need for colectomy; and (3) intolerance or adverse events (AEs).

The primary endpoint was steroid-free clinical remission, defined as no diarrhea, no haematochezia, and no need of steroids, anti-TNF alpha agents, or surgery during maintenance therapy with thiopurines. Secondary endpoints were the occurrence rate of MH in patients in steroid-free remission and long-term safety. Finally, potential clinical predictors of steroid-free clinical remission and mucosal healing were analysed.

### 2.1. Statistical Analysis

Data were described using means with standard deviation (SD) and medians with range for continuous data and percentages for discrete data. Cumulative probabilities of continuing thiopurine treatment while in remission and cumulative probability of colectomy in a patient who failed thiopurines were estimated by the Kaplan-Meier method. Associations between clinical variables and treatment efficacy (both for steroid-free remission and mucosal healing) were analysed with logistic regression analysis and expressed as odds ratio (OR) and 95% confidence intervals (95% CI). The following covariates were considered: gender, age, disease duration, disease extension, smoking habit, indication for thiopurine therapy, and concomitant aminosalicylate treatment. A two-tailed *p* value < 0.05 was regarded as statistically significant. StatsDirect statistical tools (copyright 1990–2001) were used for all calculations.

## 3. Results

### 3.1. Baseline Patients' Characteristics

One hundred and ninety-two UC patients (88 male and 104 female) receiving thiopurines as maintenance treatment were enrolled. The demographic and clinical characteristics of patients are summarised in Table 1. Median age at diagnosis was 36 years (range 16–69 years), and the median disease duration was 3.3 years (range 0–31 years). One hundred and seventeen patients (60%) had extensive colitis, and 75 patients (40%) had left-sided disease. Most patients were nonsmokers or former smokers (88%). Steroid dependency was the most common indication for thiopurine treatment (111 of 192 patients, 58%); 36 of 192 patients (19%) received thiopurines following a severe acute attack responsive to intravenous steroids, and 45 of 192 patients (23%) received thiopurines because of frequent clinical relapses despite optimized treatment with aminosalicylates.

At baseline, 148 of 192 patients (77%) were concomitantly treated with corticosteroids. More than 90% of patients received concomitant aminosalicylate maintenance.

AZA was the preferred thiopurine compared to 6MP (90% vs. 10%). All patients received thiopurines at the standard dose of 2.0–2.5 mg/kg for AZA and of 1.0–1.5 mg/kg for 6MP. For both drugs, 50 mg/day was the initial dose progressively increased to the standard dose; dose adjustment was performed during treatment according to clinical judgment. Thiopurine metabolite monitoring, as well as thiopurine methyltransferase (TPMT) activity, was not performed because it is not routinely available in clinical practice in Italy.

Endoscopic data at baseline were available for 175 of 192 patients (91.1%): 91 patients (52%) had moderate endoscopic activity classified as Mayo endoscopic subscore = 2, and 68 patients (39%) had severe endoscopic activity classified as Mayo endoscopic subscore = 3.

### 3.2. Outcomes

**Figure 1:**
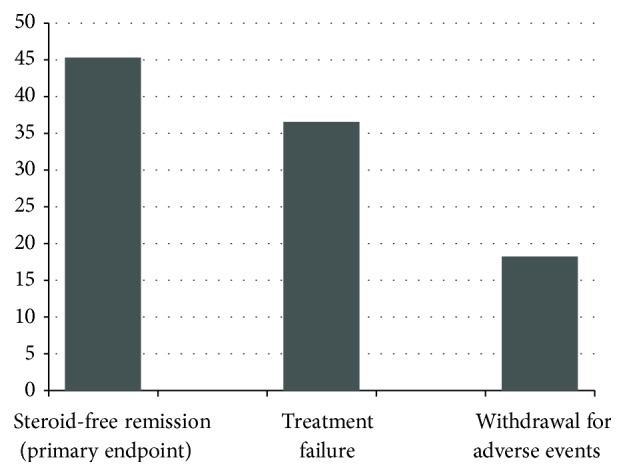
Percentage of patients achieving steroid-free clinical remission and treatment discontinuation for failure and adverse events.

The median follow-up while on thiopurine maintenance was 36 months (range 1–210 months). Participants contributed a total of 747 person-years of follow-up. Overall, 87 of 192 patients (45.3%) achieved steroid-free clinical remission within a median follow-up of 39 months (range 1–210 months). Conversely, 105 of 192 patients (54.6%) withdrew from thiopurines because of treatment failure (*n* = 70, 36.3%) or occurrence of AEs or intolerance (*n* = 35, 18.2%) (Figure 1).

Treatment failure occurred after a median follow-up of 36 months (range 3–173 months), while most patients who discontinued thiopurines for intolerance withdrew the drug within the first year (59%).

**Figure 2:**
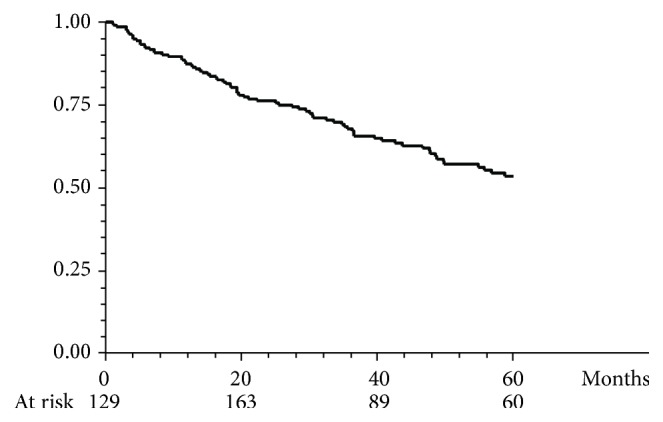
Cumulative probability of maintaining steroid-free remission while on thiopurine maintenance in the entire population.

**Figure 3:**
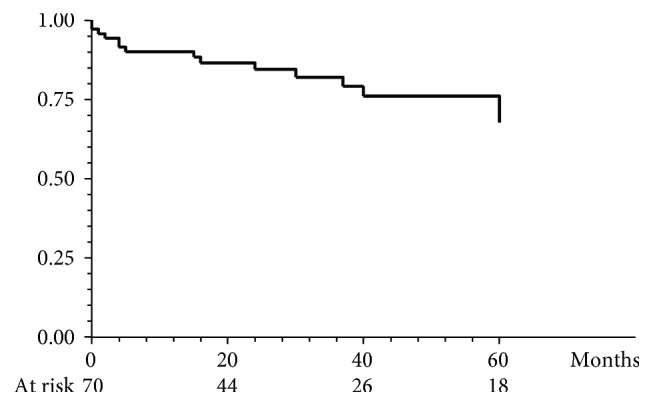
Cumulative probability of a course free of colectomy after thiopurine discontinuation for treatment failure.

The cumulative probability of maintaining steroid-free remission while on thiopurine treatment was 87%, 76%, 67.6%, and 53.4% at 12, 24, 36, and 60 months, respectively (Figure 2). Among the 87 patients who achieved steroid-free remission, 65 (73.8%) were still on thiopurine therapy at the end of the follow-up, while 22 (25%) were discontinued because of sustained remission after a median length of thiopurine treatment of 39 months (range 14–128 months). Among the 70 patients who were considered treatment failures, 57 (81.4%) received at least one course of systemic corticosteroids, 59 patients (84.2%) escalated to anti-TNF alpha agents, and 15 (21.4%) ultimately required colectomy. The cumulative probability of a course free of colectomy within 5 years after thiopurine failure was 90%, 84.4%, 82.0%, and 67.6% at 12, 24, 36, and 60 months, respectively (Figure 3).

**Figure 4:**
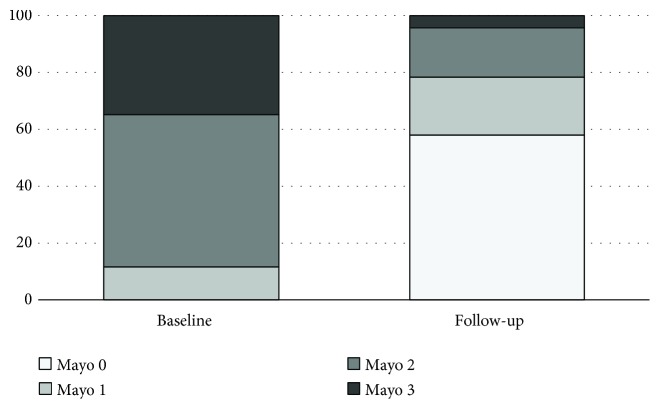
Endoscopic activity according to the Mayo endoscopic subscore at baseline and during follow-up in the subgroup of 69 of 87 responders who underwent endoscopy both before starting thiopurines and during follow-up. Mucosal healing (Mayo endoscopic subscore = 0) was achieved in 58% of patients.

As far as MH is concerned, data are available for a subgroup of 69 of 87 responders, whose baseline and follow-up endoscopy data were available. Follow-up endoscopies were performed after a median time of 18 months (range 5–96 months) after starting thiopurines, according to clinical judgment. Endoscopic activity, expressed as Mayo endoscopic subscore, at baseline and during follow-up is shown in Figure 4. Overall, 40 of 69 patients (57.9%) achieved complete MH while on thiopurine maintenance (Mayo endoscopic subscore = 0).

A logistic regression analysis was performed to explore possible clinical predictors of treatment success. None of the clinical variables included in the model was associated with the probability of steroid-free remission (Table 2) or mucosal healing (data not shown).

### 3.3. Safety

A total of 45 patients experienced at least one AE related to thiopurine exposure. Overall, 35 patients discontinued thiopurines because of AEs or intolerance. The description and frequency of all AE events in our cohort are reported in Table 3. Gastrointestinal intolerance (including nausea and vomiting) occurred in 13 patients (29%). In 3 patients, switch to 6MP was attempted without success. Thirteen patients (29%) experienced leukopenia (a white blood cell count < 3000/mm), and among them, 10 needed drug discontinuation. Elevation of serum transaminases (more than 2–3 times the upper limit of normal) was recorded in 6 patients (13%), and 5 patients were consequently discontinued. No one developed chronic liver disease. In 5 patients (11%), elevation of serum pancreatic enzymes occurred, but only two patients (4%) developed acute pancreatitis requiring hospital admission. Infections were recorded in 14 patients (31%), but only three of them (2 cases of *Listeria monocytogenes* infection and 1 case of Cytomegalovirus colitis) were considered severe and required hospitalization. Two patients (4%) developed malignancies (1 anal cancer and 1 gastric cancer).

## 4. Discussion

Although thiopurines are widely used as a maintenance treatment in UC and are considered at least as effective as in CD patients [19], controversy still exists regarding their efficacy in maintaining remission in the long term [15]. Evidence-based data supporting the efficacy of AZA and 6MP in UC are limited, and the main evidence comes from observational studies, mainly retrospective. Observational studies report substantial variability in effectiveness of thiopurines in UC, ranging from 40% to 70% [20–26]. However, significant heterogeneity across studies, methodological limitations, small sample size, variable length of follow-up, and different endpoint definitions highlight the uncertainty of the available data.

Our study focuses on the long-term outcome of thiopurine treatment in UC patients in a real-life setting. Although the main limitation of our study is its retrospective design, the large number of patients included and the consistent length of follow-up (760 person-years) are the main strengths. Moreover, we report data addressing MH in a large subgroup of patients, and this represents a peculiarity of our study because thiopurine-induced MH has not been extensively studied and it is usually not assessed in most observational studies [24–26]. Another strength of our study is the strict definition of steroid-free clinical remission, our primary endpoint, that is, the absence of diarrhea and blood in stools, without need of any escalation of therapy, including steroids, anti-TNF alpha agents, or surgery. As previously reported, stool frequency and rectal bleeding alone provide reasonable estimates of disease activity as well as the Mayo scoring system, commonly used in RCTs [27].

MH has been strictly defined as a Mayo endoscopic subscore = 0. Although in several RCTs and cohort experiences MH is usually defined as a Mayo endoscopic subscore ≤ 1 [28], recent observations suggest that there is an improved long-term outcome in patients achieving complete MH (Mayo subscore = 0) compared to patients achieving partial MH (Mayo subscore = 1) [29]. Finally, a survival regression model has been performed to explore possible predictors of sustained efficacy of thiopurines.

Overall results show that approximately 45% of UC patients receiving thiopurines achieve steroid-free remission, 37% fail to respond to thiopurines and need escalation therapy, and less than 20% discontinue the drug because of AEs or intolerance.

Our results suggest a favourable profile of thiopurines in UC in terms of long-term efficacy and safety and are comparable to data recently reported by Sood et al. In their cohort of 255 UC patients, after a median follow-up of 30 months, 60.4% achieved remission, approximately 20% required escalation of therapy, and 30% experienced AEs resulting in thiopurine discontinuation [26]. Other smaller observational studies report comparable results [24, 25]. The probability of achieving steroid-free clinical remission is unpredictable; logistic regression analysis failed to identify any clinical predictor of treatment success confirming previous observations [26]. However, it is interesting to note that early introduction of thiopurines, within the first year after diagnosis, was associated with a reduced probability of achieving steroid-free remission although the data is not statistically significant. We can speculate that patients who require early introduction of thiopurines have a more severe disease onset and a more aggressive early clinical course leading to a worse outcome.

Data concerning endoscopic remission are not available in recently published large series [26]. We have studied the occurrence rate of MH in a subgroup of patients who achieved steroid-free remission and who underwent colonoscopy at baseline and after a median of 12 months (range 1–132) after starting thiopurines. Complete endoscopic remission, defined as a Mayo endoscopic subscore = 0, was observed in more than 50% of patients. In recent years, targeting MH is an emerging therapeutic endpoint in the management of UC [16, 30]. MH has been associated to a more favourable outcome in terms of reduction of clinical relapse, steroid needs, hospitalizations, colorectal cancer, and surgery [31]. Although it is commonly accepted that thiopurines are able to induce MH, this effect is slow, the occurrence rate of MH in thiopurine-treated UC has not been systematically investigated, and few data are available. In a recent multicenter retrospective French study on 80 UC patients receiving thiopurine monotherapy, MH (defined as a Mayo endoscopic subscore ≤ 1 and Ulcerative Colitis Endoscopic Index of Severity (UCEIS) < 2) was observed in 43.7% after a mean follow-up of 38 ± 31 months after thiopurine introduction [32]. These findings are similar to our observations.

AEs requiring withdrawal from therapy occurred in 18.2% of patients, a figure similar to that reported in other observational studies [19–24]. However, in other cohort studies, some of which include both CD and UC patients, the occurrence rate of AEs leading to thiopurine discontinuation may be as high as 25–40% [23, 26, 33–35]. In our study, the most common causes of AZA cessation were gastrointestinal symptoms, despite the fact that a slow dose escalation approach was adopted in most patients. A switch to 6MP was attempted in a minority of patients. Myelotoxicity and hepatotoxicity requiring drug discontinuation occurred in about 5% and 3% of patients, respectively. We have no data on TPMT activity and serum thiopurine metabolite concentrations: monitoring metabolites is not a routine practice in Italy, and this approach is not available in most of the hospitals.

In conclusion, in our real-life experience on a large cohort of UC patients, thiopurines are effective for maintaining long-term steroid-free clinical remission and for inducing MH. No predictors of long-term benefit could be identified. Less than 20% of patients discontinue the drug because of AEs or intolerance supporting a favourable benefit/risk profile of thiopurines in UC.

## Figures and Tables

**Table 1:** Demographic and clinical characteristics of patients.

	Patient *n* = 192
*Gender n (%)*	
Female	104 (54.1)
Male	88 (45.9)
*Age, years*	
Median (range)	36 (16–69)
*Disease duration, years*	
Median (range)	3.3 (0–31)
*Disease extension n (%)*	
Extensive colitis	117 (60)
Left-sided colitis	75 (40)
*Mayo endoscopic subscore *∗*n (%)*	
Mayo 1	16 (9)
Mayo 2	91 (52)
Mayo 3	68 (39)
*Smoking habit n (%)*	
Yes	23 (12)
No/former smoker	169 (88)
*Indication for starting thiopurines n (%)*	
Steroid dependency	111 (58)
Maintenance after a severe attack	36 (19)
Frequent relapses	45 (23)
*Cotreatment with mesalazine n (%)*	175 (91.1)
*Duration of thiopurine therapy, months*	
Median (range)	36 (1–210)

**Table 2:** Logistic regression analysis of predictors of steroid-free clinical remission.

Covariates	OR	95% CI	*p* value
*Gender* (female vs. male)	1.12	0.63–1.98	ns
*Age* (>40 years vs. <40 years)	0.77	0.42–1.40	ns
*Extension* (distal colitis vs. extensive colitis)	0.85	0.48–1.49	ns
*Smoking habit* (yes vs. no/ex)	1.17	0.54–2.53	ns
*Starting thiopurine* (within 1 year vs. >1 year after diagnosis)	0.48	0.23–1.01	ns
*Indication for thiopurine Tx* (no steroid dependency vs. steroid dependency)	1.51	0.83–2.73	ns
*Concomitant aminosalycilates* (no vs. yes)	1.87	0.80–4.39	ns

**Table 3:** Adverse events related to thiopurine exposure.

Type of event	*n* = 45 (%)
Nausea and vomiting	13 (29)
Leukopenia	13 (29)
Elevation of transaminases	6 (13)
Elevation of pancreatic enzymes	3 (6)
Acute pancreatitis	2 (4)
Myalgia	1 (2)
Alopecia	1 (2)
Infections	14 (31)
Serious infections	3 (6)
Malignancy	2 (4)

## Data Availability

The general dataset is available upon request writing to the corresponding author, Daniela Pugliese.
